# Comparison of perioperative flurbiprofen axetil or celecoxib administration for pain management after total-knee arthroplasty: A retrospective study

**DOI:** 10.1097/MD.0000000000012391

**Published:** 2018-09-14

**Authors:** Xia Xiao, Qing Zhang, Zhengxiao Ouyang, Xiaoning Guo

**Affiliations:** a Department of Orthopedics; b Clinical Nursing Teaching and Research Section, The Second Xiangya Hospital, Central South University, Changsha, Hunan Province, China.

**Keywords:** analgesia, celecoxib, flurbiprofen axetil, postoperative pain, preemptive analgesia, total-knee arthroplasty

## Abstract

Nonsteroidal anti-inflammatory drugs (NSAIDs) are recommended for multimodal postoperative pain management. The purpose of this study was to evaluate the postoperative pain relief, time to ambulation, and opioid-sparing effects of flurbiprofen axetil (FA) and celecoxib (CX) after total-knee arthroplasty (TKA) surgery.

A convenience sample of 300 patients was obtained using a retrospective chart review of patients who underwent TKA and received FA or CX or saline (SA) as control. Institutional review board approval was obtained, and 300 charts of patients who received TKA were reviewed. Visual analog scale (VAS) pain scores up to 6 months postoperatively, opioid requirements, range of knee motion, adverse effects, and length of hospital stay are recorded. Data were analyzed using the Pearson Chi-square where appropriate or the Fisher exact test, and all continuous variables were examined using a Wilcoxon rank test.

The results of the study showed no significant differences between the 3 groups for the age, gender, American Society of Anesthesiologists class, number of patients who underwent knee surgery, weight, height, and operation duration. Patients in FA and CX demonstrated significantly reduced pain scores and less morphine consumption at rest and active motion compared to SA in 24 hours after surgery, with lower scores and less opioid requirements in the FA group. However, after 48 hours postoperatively, there are no significant differences between these groups.

Intravenous application of 1 mg/kg flurbiprofen axetil twice a day and 200 mg celecoxib once a day improved analgesia and decreased morphine consumption following TKA. When the 2 active drugs were compared, it was found that flurbiprofen axetil was superior to celecoxib in terms of short-term analgesic efficacy and opioid consumption.

## 1 Introduction

Total-knee arthroplasty (TKA) has become most common therapy for patients with severe knee functional impairment, which can significantly relieve pain, restore joint function, and improve quality of life.^[1]^ The number of patients undergoing TKA has increased steadily each year, and this trend will continue in the foreseeable future due to the aging of the population.^[2]^ Postoperative pain after total-joint arthroplasty has remained a serious problem, which prolongs hospitalization and functional recovery. Therefore, appropriate pain management is necessary. Opioids are the most powerful and effective drugs for pain relief, and have generally been used for pain control after TKA.^[3]^ However, common side effects, such as sedation, dizziness, nausea, vomiting, constipation, physical dependence, tolerance, and respiratory depression, are serious problems with these medications.^[4]^ Thus, the combination of nonopioid and opioid analgesics, known as multimodal analgesia, has become a cornerstone in the treatment of postoperative pain.

Nonsteroidal anti-inflammatory drugs (NSAIDs) and opioid drugs are known to possess synergistic analgesic effects. Pretreatment with NSAIDs enhances analgesic effect and reduce the consumption of opioids in patients undergoing many kinds of surgery.^[5–8]^ Flurbiprofen axetil (FA), which is incorporated in lipid microspheres, is a NSAID with high affinity to the site of surgical incision.^[9]^ Celecoxib (CX) is a selective cyclooxygenase-2 (COX-2) inhibitor shown to be as effective as traditional NSAIDs as an analgesic for acute postoperative pain and has fewer gastrointestinal side effects than traditional NSAIDs.^[7]^ There are a few reports compare the effectiveness of preoperative flurbiprofen axetil and celecoxib on postoperative analgesic and perioperative opioid consumption after TKA. Thus, the aim of this retrospective study was to compare the efficacy of intravenous flurbiprofen axetil 1 mg/kg with that of orally administration of 100 mg celecoxib and placebo (PB) as a constituent of multimodal agalgesia after TKA.

## 2 Materials and methods

We retrospectively analyzed all patients included in a prospective data bank from January 1, 2013 to January 1, 2016 underwent primary TKA by the same surgeon who had been in practice for over 5 years in a private practice model in our orthopedic institution. Patients with endocrine disorders, severe hepatic and renal diseases, neuropathies, bleeding disorders, preexisting gastric ulcers, gastritis, and history of gastrointestinal bleeding, dementia, cooperation disability and sensitivity to FA, CX, or morphine were not included. All written informed consent was obtained from each patient or family member before inclusion in this study. The study protocol was approved by the institutional review board and Ethics Committees of The Second Xiangya Hospital and Central South University. The conventional CX was used in 100 consecutive patients undergoing TKA (group A) from January 2014 to June 2015. The FA was subsequently performed in 100 additional consecutive patients (group B) from July 2015 to December 2016, and none was used in 100 patients during January 2013 to December 2013 (group C). Personal and clinical data of all patients including age, gender, American Society of Anesthesiologists (ASA) class, number of patients who underwent knee surgery, weight, height, and operation duration, visual analog scale (VAS), opioid requirements, range of knee motion, adverse drug reactions (ADRs), length of hospital stay was collected by a research assistant and reviewed retrospectively. There were 68 females and 32 males in group A. Group B was composed of 59 females and 41 males and group C was 65 females and 35 males. The average age of group A was 64 years (range 55–73) and 60 years for group B (range 52–68) and 61 years for group C (range 54–70). The preoperative diagnosis in group A was osteoarthritis in all patients except 1 diagnosis of posttraumatic arthritis. In groups B and C osteoarthritis was again most common with only 1 diagnosis of rheumatoid arthritis and 2 posttraumatic arthritis.

Based on a multidisciplinary approach, patients receiving multidisciplinary pain program were divided into 3 groups.

*Group A* (FA): Patients (n = 100) had 1 mg/kg intravenous flurbiprofen axetil twice a day: first dose was applied 15 min before surgery, and the second injection was administered 1 hours after surgery.

*Group B* (CX): Patients (n = 100) had 200 mg orally once a day: first dose was applied 12 hours before the surgery, and the second dose was administered 12 hours later.

*Group C* (PB): Patients (n = 100) had no analgesic treatment except for morphine in recovery room and patient controlled analgesia (PCA).

### 2.1 Postoperative analgesia

In the recovery room patients were given 0.05 mg/kg intravenous morphine as required to stabilize their pain and at the same time for postoperative analgesia use of the PCA device was started (intravenous [IV] morphine 0.01 mg/kg bolus dose and 10 min lockout time).

### 2.2 Evaluation parameters

During the postoperative period, the degree of subjects’ pain was evaluated with a 0 to 100 mm VAS. Scores were obtained at 1, 2, 4, 6, 8, 12, 24, 48 hours and 3, 6 months after surgery at rest and during movement. Total-morphine consumption via PCA device, ADRs in the first 24 hours postoperatively as well as length of hospital stay was recorded. The clinical and radiologic follow-up to determine range of knee motion in 6 and 12 months postoperatively was assessed.

### 2.3 Statistical analysis

Differences among groups for the VAS were tested using an analysis of variance (ANOVA). Others differences among groups were analyzed using an ANOVA or Kruskall–Wallis, Chi-square as appropriate. Differences in group demographic characteristics were tested by ANOVA or contingency table Chi-squared test for categorical measures. A level of *P* < .05 was considered statistically significant.

## 3 Results

With the approval of local ethics committee and signed informed consent of the patients, 300 ASA I–III patients underwent elective unilateral knee replacement surgery were included in the study. A comparison of groups with respect to age, sex, body weight, and operative characteristics showed no statistically significant difference (Table 1).

**Table 1:**
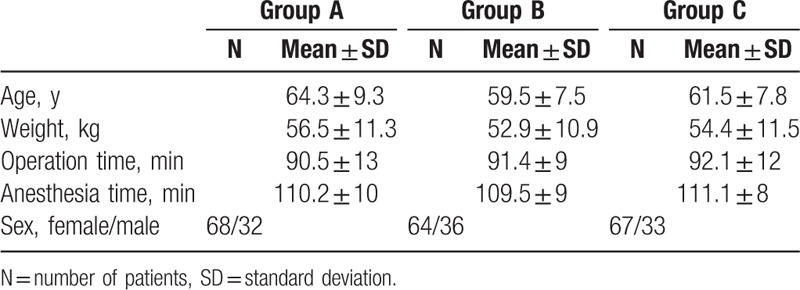
Patient characteristics (mean ± SD).

### 3.1 VAS scores at rest

**Figure 1:**
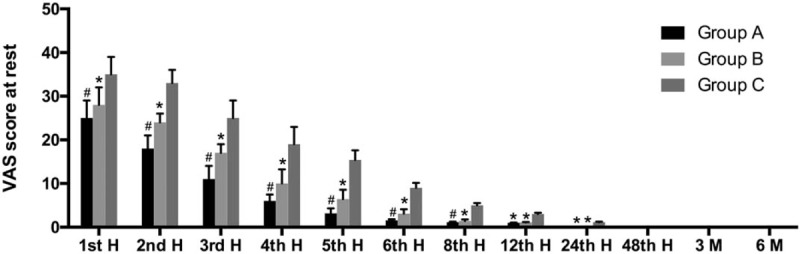
Visual analog scale (VAS) scores at rest (mean ± standard deviation). ∗*P* < .05 when compared with group C. ^#^*P* < .05 when compared with groups B and C.

At the early postoperative period, the rest VAS scores of all groups were high and decreased in time; the reduction of VAS scores of patients in group A was faster, while it was slowest in patients in group C. Patients in groups A and B had significantly lower VAS scores than patients in group C at 1 to 24 hours postoperatively at rest. Also, the VAS values of group B patients were significantly higher than group A patients at 1 to 8 hours at rest. There was no significant difference between 3 groups in VAS score after 48 hours (Fig. 1).

### 3.2 VAS scores during movement

**Figure 2:**
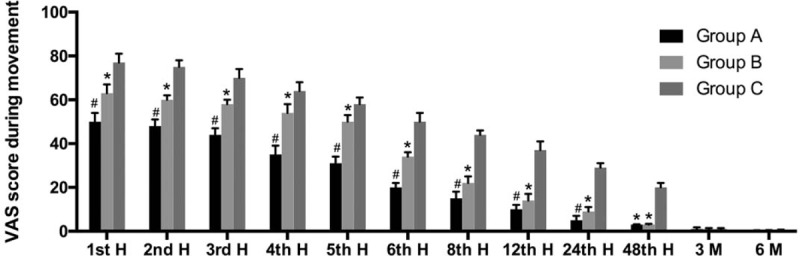
Visual analog scale (VAS) scores during movement (mean ± standard deviation). ∗*P* < .05 when compared with group C. ^#^*P* < .05 when compared with group B.

At the early postoperative period, VAS scores of all the groups during movement were high and decreased in time; the reduction of VAS scores was faster in patients in group A, while it was slowest in group C. Patients in group A had significantly lower VAS values than patients in group B during movement only in 24 hours postoperatively; however, there were no significant difference in the study period after 24 hours. Patients in groups A and B had significantly lower VAS values than patients in group C at 1 to 48 hours (Fig. 2).

### 3.3 Morphine consumptions of the groups

Patients in group A used 5.0 mg (range 2.5–7.5 mg) morphine while patients in group B used 7.5 mg (range 2.5–7.5 mg) and patients in group C used 10.0 mg (range 7.5–12.5 mg) (group A vs group C *P* < .05, group B vs group C *P* < .05 and group A vs group B *P* < .5) (Table 2).

**Table 2:**
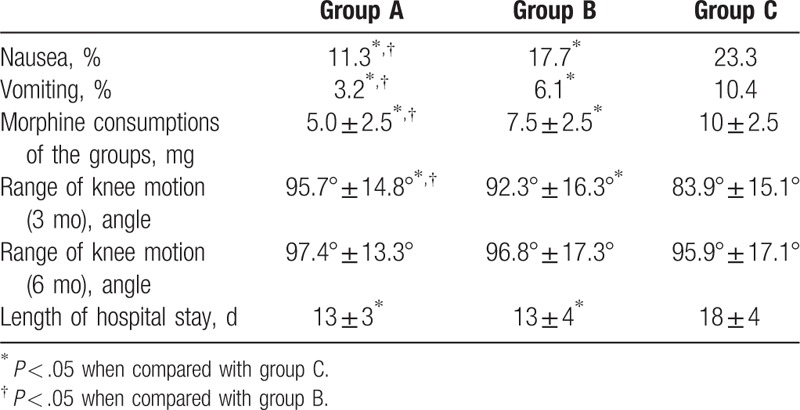
Opioid side effects and operative knee outcomes.

### 3.4 Range of knee motion

Range of knee motion was assessed at 3 and 6 months postoperatively. The flexion angles of patients in groups A and B were 95.7° ± 14.8° and 92.3° ± 16.3° at 3 months postoperatively, which were significantly better than group C (83.9° ± 15.1°) (*P* < .05). However, there was no significant difference in flexion angle between groups A, B, and C at 6 months after surgery (Table 2).

### 3.5 Adverse drug reactions

Patients in groups A (11.3%) and B (17.7%) have significantly lower incidence of nausea than group C (23.3%), with much lower in group A. Similar results were also seen in vomiting (3.2% in group A vs 6.1% in group B vs 10.4% in group C) (*P* < .05) (Table 2).

### 3.6 Length of hospital stay

Patients in groups A and B have significantly shorter hospitalization than group C (23.3%), but there was no significant difference between groups A and B (13 ± 3 days in group A vs 13 ± 4 in group B vs 13 ± 4 in group C) (*P* < .05) (Table 2).

## 4 Discussion

The TKA is accepted as an effective operation for end-stage osteoarthritis and other severe knee disease.^[10]^ However, TKA is associated with a considerable amount of postoperative pain.^[11]^ Sommer et al^[12]^ found that 20% to 71% of patients who had TKA suffer from moderate to severe pain during the first 1 to 4 days. Failure to adequately control pain after TKA may induce pathophysiologic responses, which increase anxiety, disrupt sleep, or result in a higher prevalence of unsatisfactory postoperative results.^[13]^ In addition, negative clinical outcomes included deep vein thrombosis, pulmonary embolism, and poor wound healing also resulting from ineffective postoperative pain management.^[6]^ Therefore, sufficient postoperative pain management is considered mandatory in modern TKA.^[1]^

Numerous techniques have been proposed to minimize postoperative pain after TKA. Opioid drugs are used as first line drugs; but because of serious side-effects, they are not used in adequate doses to treat moderate to severe pain.^[14]^ Preemptive analgesia, a new concept to enhance the postoperative analgesia, is to give a first dose of analgesics before pain stimulation has been considered as multimodal analgesia. Previously, prophylactic administration of NSAIDs, as a multimodal analgesia, has been found to reduce postoperative VAS pain score, opioid analgesic consumption, and has potential benefits of earlier independent ambulation and active ROM of the knee after TKA surgery.^[7,8]^ FA and CX are the 2 of the most popular NSAIDs clinically used in orthopedics major surgery. Thus, it is necessary to verify and compare the effectiveness of administration of NSAIDs during the postoperative period as part of multimodal analgesia. To date, however, no comparative data are available.

Our results showed that the VAS score at rest and during movement of patients under TKA who receive FA as preemptive analgesia is lower than who receive CX, which indicates the combination of FA and opioid might exert stronger effect of pain relieving. However, after 8 hours postoperatively, there was no significant difference in VAS score between FA and CX in resting activity and after 24 hours similar results could be seen in patients’ movement. Comparing to CX which administrated orally, FA is delivered intravenously, which might have the benefits of fast absorption and high bioavailability. In addition, FA designed to target to surgical incision, high local concentration might be another reason for stronger analgesic effect. Due to the similar results of medium- and long-term analgesic effect of FA and CX, the unique advantage of CX, such as lower gastrointestinal toxicity and ease of administration, support the use of CX for long-term postoperative pain control.

Opioid is useful and effective for postsurgical pain control. However, the incidence rates of postoperative nausea and vomiting as its side effects could be increased with large amounts of opioid. In addition, some patients would be easily addicted to opioid medications used after TKA.^[15]^ Thus it is very important to reduce opioid consumption after surgery. By taking FA and CX, opioid consumption and its side effects were reduced. As expectations, with stronger effect of analgesia, FA offers advantage on aspect of reducing opioid consumption and its side effects.

When postoperative pain is reduced, the range of knee motion with accelerated physical recovery might be improved and the duration of hospital stay might be reduced. In our study, both FA and CX were shown to improve pain control, and less pain can lead to more extensive activities, which may be the reason for the improvement of knee function. However, after 6 months postoperatively, this difference could not be seen, which might because patients with lower function of knee might seek for rehabilitation, and this could hardly be recorded in our data bank.

The NSAIDs are associated with many adverse effects, including reducing platelet aggregation, renal and gastrointestinal mucosal injury.^[7]^ In our present study, there were no adverse effects found in any of the involved patients, which might be due to the relatively short period of FA and CX administration in the present study.

Though some clinical studies have conflicting results regarding the efficacy of preemptive analgesia,^[16]^ similar to other studies,^[7,9]^ our present study suggested preoperative administration of FA and CX had preemptive analgesia effects. Researchers argue that almost all the analgesic studies were published by anesthetists, and some important details usually considered by orthopedics, such as surgical approach, technique of incision, and type of implant were not reported in those papers.^[15]^ All of these factors have the ability to change the degree of postoperative pain, and we taken them into account in our studies. Because TKA based on primary osteoarthritis is a standard operation procedure^[17–21]^ and all patients are treated by the senior surgeon, no influence on the results exists by different operation techniques.

However, there are still several limitations in the present study. Retrospective studies that use administrative data may contain unintended bias. Retrospective study design does not allow for comprehensive data collection directly related to the study question. Psychosocial characteristics, educational background, and preoperative pathology of the patients that were not controlled, and it is also not well known in our study whether pain relief attenuates the postoperative systemic inflammatory response. It is also not known that the doses of active drugs used are equipotent or not; but the doses were chosen according to the manufacturers’ recommendations. These drugs are not compared with each other in major orthopedic surgery and further studies are needed comparing different doses of these drugs. For the highest scientific excellence, a double-blinded, randomized controlled study with a fixed dose of selective pain killers would be preferable and further studies should consider those open points.

## 5 Conclusion

The final goal of effective pain treatment is to ensure the lowest pain intensity during rest and ambulation and the lowest need for supplemental opioids in a majority of patients. Our pooled data showed that FA and CX both could exert ideal analgesic effects and decrease postoperative opioid consumption in patients undergoing TKA. The most important finding of the present study was that IV FA 1 mg/kg twice a day (first dose 15 minutes before surgery and second dose at 1 hour postoperatively) provided a better pain relief than CX 200 mg once a day after TKA under general anesthesia. This will help to provide clinical management status for patients with TKA surgery.

## Author contributions

**Conceptualization:** Xia Xiao, Zhengxiao Ouyang, Xiaoning Guo.

**Data curation:** Qing Zhang, Xia Xiao.

**Investigation:** Xia Xiao.

**Methodology:** Qing Zhang, Zhengxiao Ouyang.

**Supervision:** Xiaoning Guo.

**Validation:** Qing Zhang.

**Writing – original draft:** Zhengxiao Ouyang.

**Writing – review & editing:** Xiaoning Guo.
